# Supplementary figures and images for: GeneCOCOA: Detecting context-specific functions of individual genes using co-expression data
Source: PLoS Comput Biol. 2025 Mar 31;21(3):e1012278. doi: 10.1371/journal.pcbi.1012278 (PMC11964461; doi:10.1371/journal.pcbi.1012278)

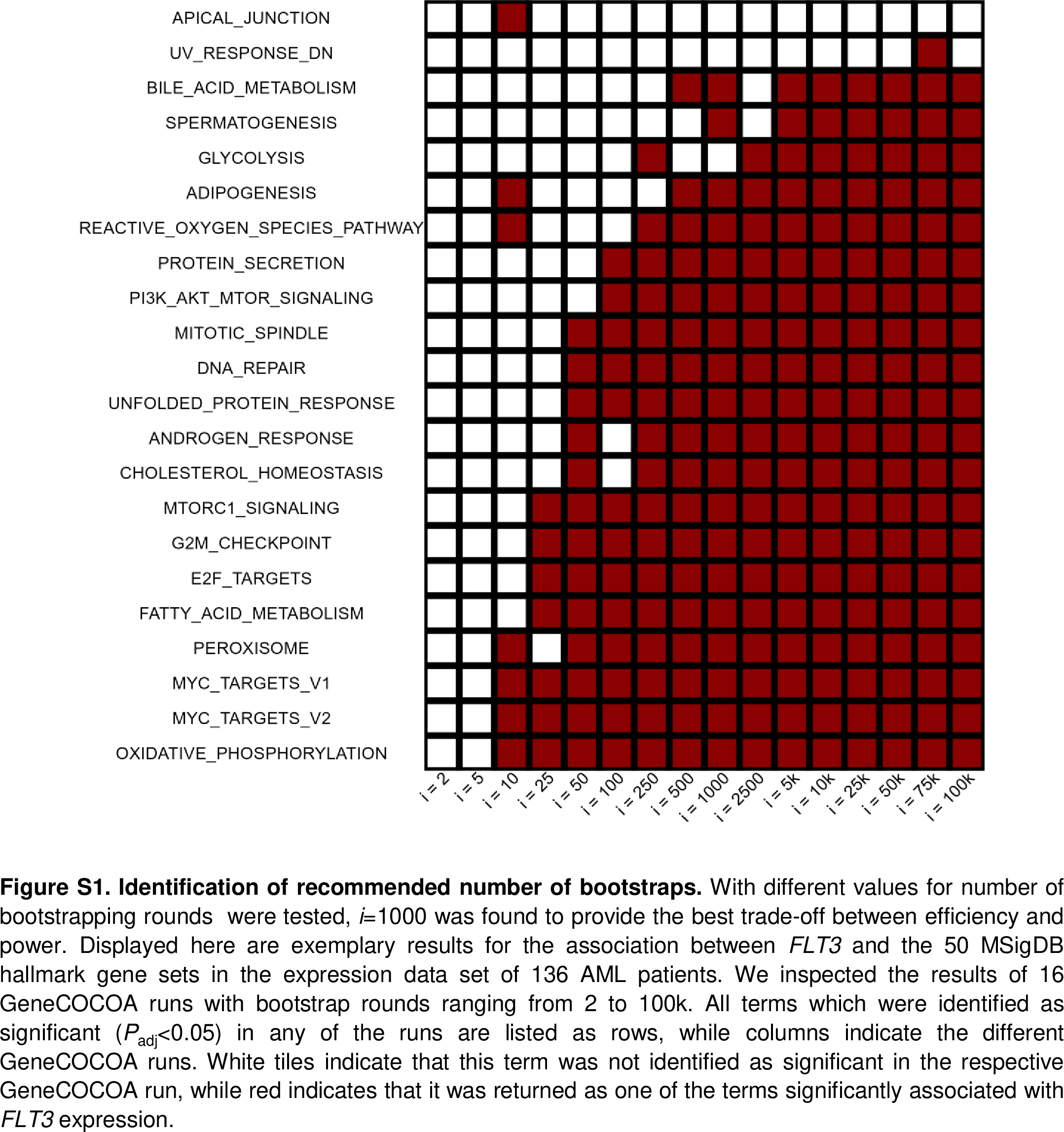

Supplement: S1 Fig — With different values for number of bootstrapping rounds were tested, i = 1000 was found to provide the best trade off between efficiency and power. Displayed here are exemplary results for the association between FLT3 and the 50 MSigDB hallmark gene sets in the expression data set of 136 AML patients. We inspected the results of 16 GeneCOCOA runs with bootstrap rounds ranging from 2 to 100,000. All terms which were identified as significant Padj in any of the runs are listed as rows, while columns indicate the different GeneCOCOA runs. White tiles indicate that this term was not identified as significant in the respective GeneCOCOA run, while red indicates that it was returned as one of the terms significantly associated with FLT3 expression. (TIF) [file pcbi.1012278.s001.tif]

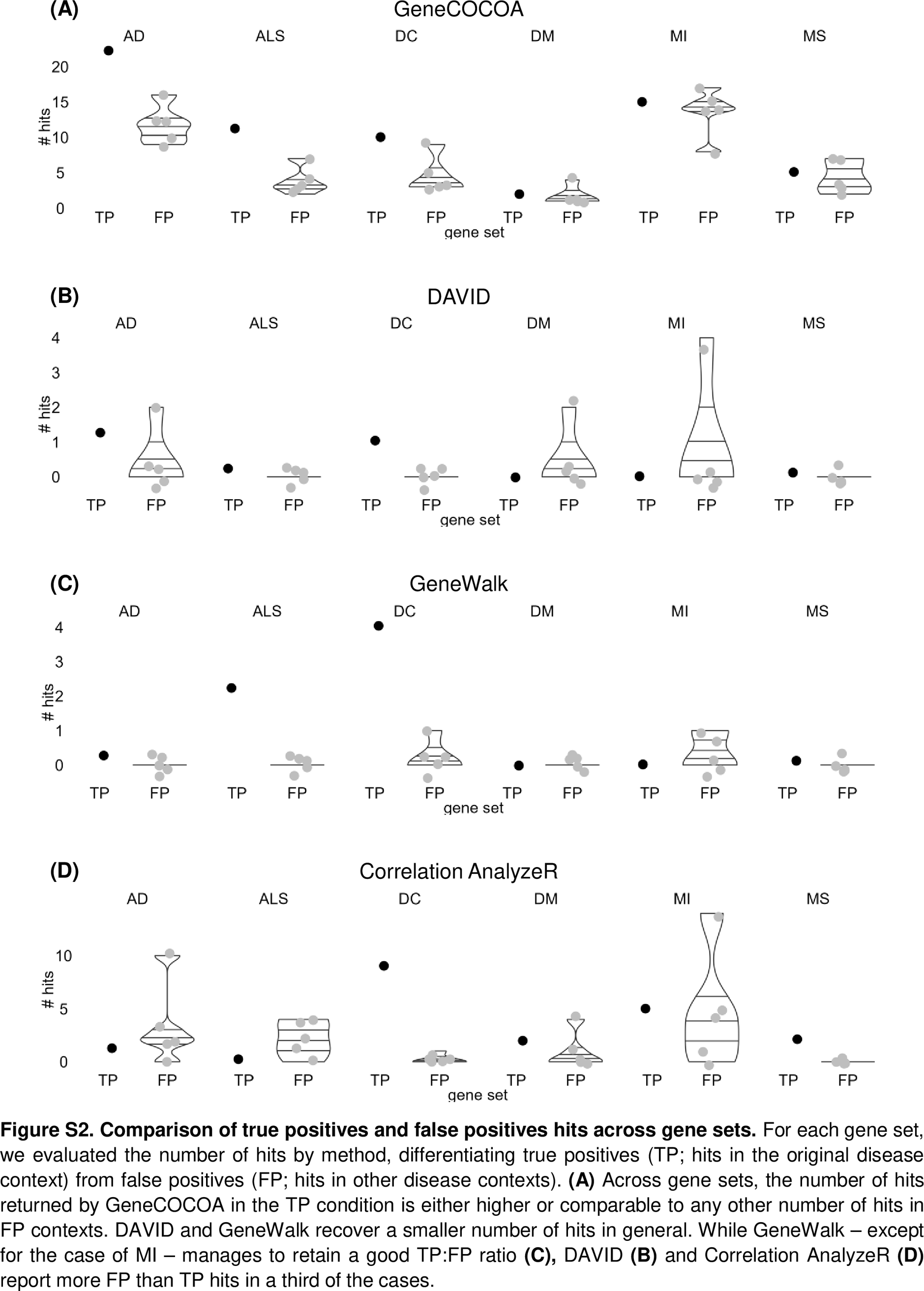

Supplement: S2 Fig — For each gene set, we evaluated the number of hits by method, differentiating true positives (TP hits in the original disease context) from false positives (FP hits in other disease contexts) (A) Across gene sets, the number of hits returned by GeneCOCOA in the TP condition is either higher or comparable to any other number of hits in FP contexts. DAVID and GeneWalk recover a smaller number of hits in general. While GeneWalk – except for the case of MI – manages to retain a good TP:FP ratio (C) DAVID (B) and Correlation AnalyzeR (D) report more FP than TP hits in a third of the cases. (TIF) [file pcbi.1012278.s002.tif]

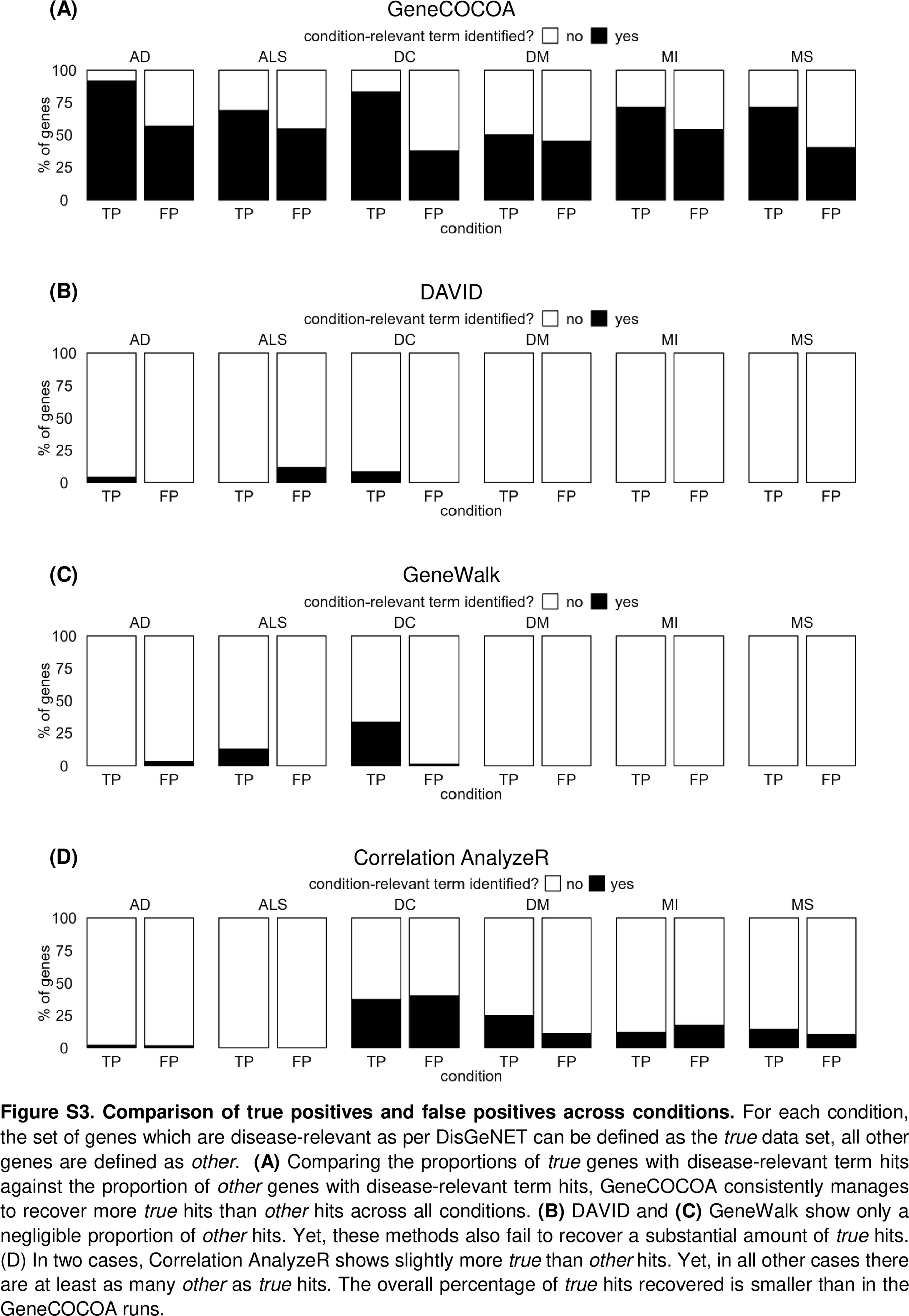

Supplement: S3 Fig — For each condition, the set of genes which are disease relevant as per DisGeNET can be defined as the true data set, all other genes are defined as other. (A) Comparing the proportions of true genes with disease relevant term hits against the proportion of other genes with disease relevant term hits, GeneCOCOA consistently manages to recover more true hits than other hits across all conditions. (B) DAVID and (C) GeneWalk show only a negligible proportion of other hits. Yet these methods also fail to recover a substantial amount of true hits. (D) In two cases, Correlation AnalyzeR shows slightly more true than other hits. Yet, in all other cases there are at least as many other as true hits. The overall percentage of true hits recovered is smaller than in the GeneCOCOA runs. (TIF) [file pcbi.1012278.s003.tif]

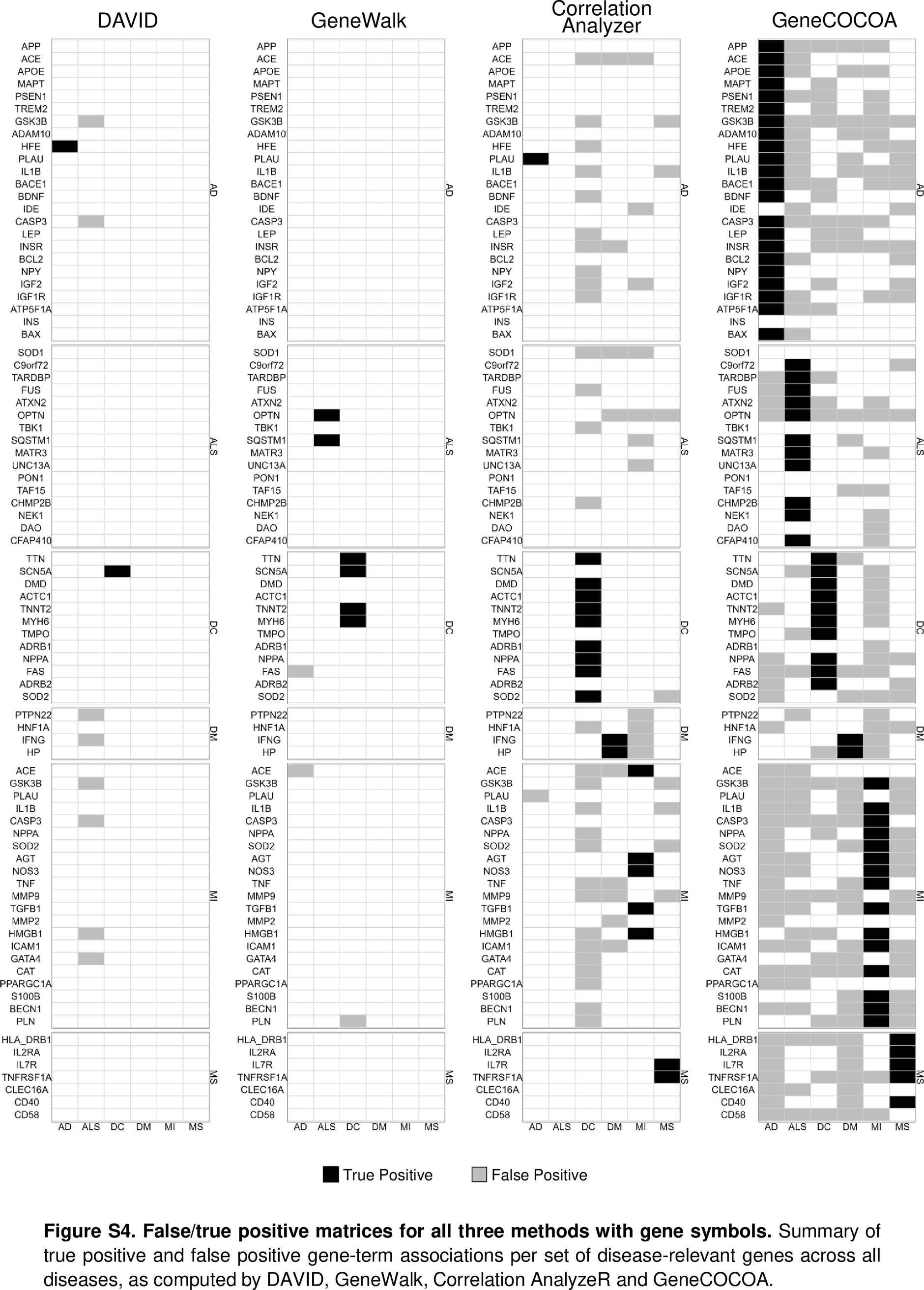

Supplement: S4 Fig — Summary of true positive and false positive gene term associations per set of disease relevant genes across all diseases, as computed by DAVID, GeneWalk, Correlation AnalyzeR and GeneCOCOA. (TIF) [file pcbi.1012278.s004.tif]

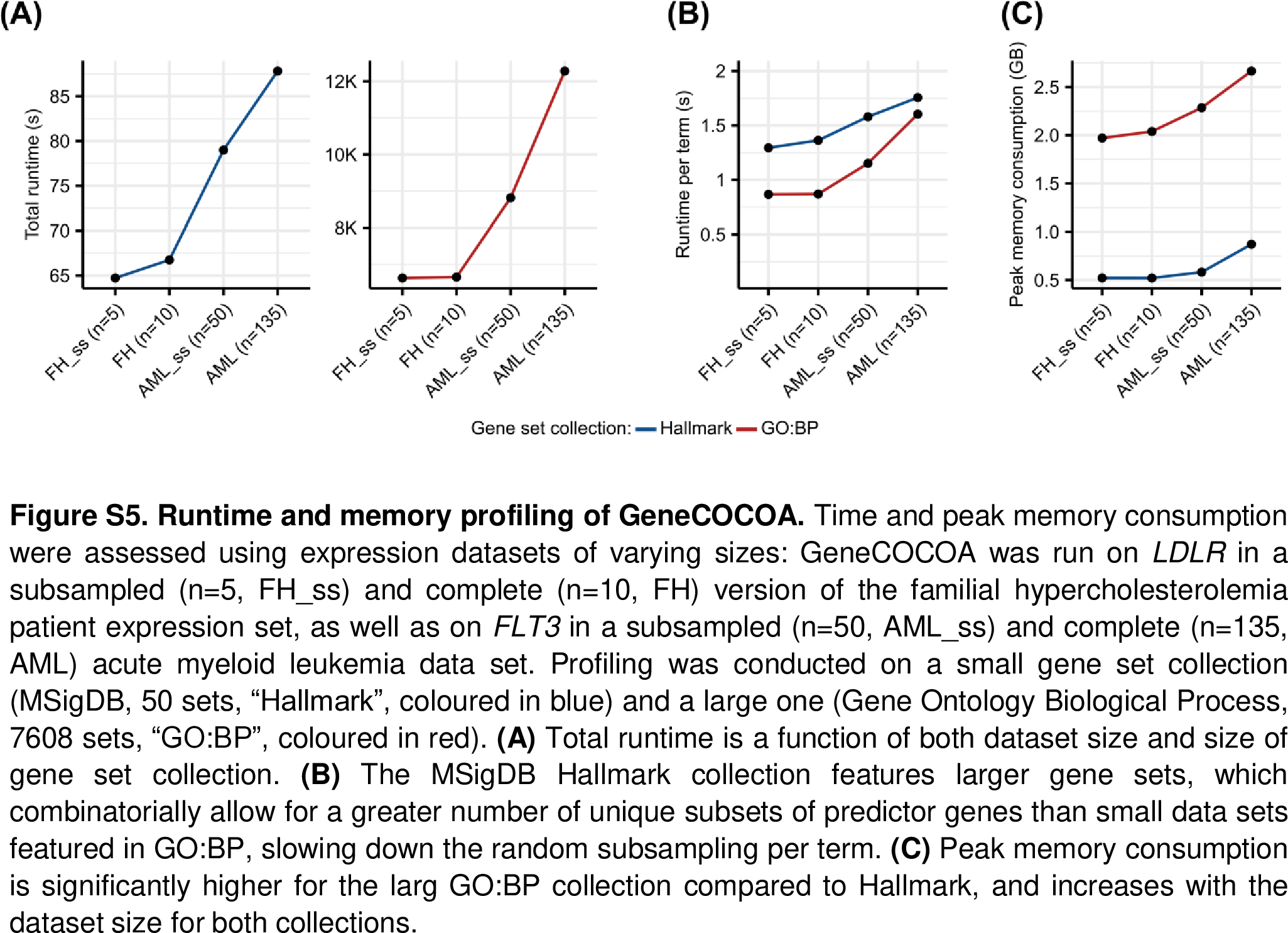

Supplement: S5 Fig — Time and peak memory consumption were assessed using expression datasets of varying sizes: GeneCOCOA was run on LDLR in a subsampled (n = 5, FH_ss) and complete (n = 10, FH) version of the familial hypercholesterolemia patient expression set, as well as on FLT3 in a subsampled (n = 50, AML_ss) and complete (n = 135, AML) acute myeloid leukemia data set. Profiling was conducted on a small gene set collection (MSigDB, 50 sets, “Hallmark”, coloured in blue) and a large one (Gene Ontology Biological Process, 7608 sets, “GO:BP”, coloured in red). (A) Total runtime is a function of both dataset size and size of gene set collection. (B) The MSigDB Hallmark collection features larger gene sets, which combinatorially allow for a greater number of unique subsets of predictor genes than small data sets featured in GO:BP, slowing down the random subsampling per term. (C) Peak memory consumption is significantly higher for the larg GO:BP collection compared to Hallmark, and increases with the dataset size for both collections. (TIF) [file pcbi.1012278.s005.tif]
